# GENERATIVE AI IN GERONTOLOGY: CENTERING STAKEHOLDERS’ VOICES

**DOI:** 10.1093/geroni/igae098.1917

**Published:** 2024-12-31

**Authors:** George Demiris, Hilaire Thompson

**Affiliations:** Chair; Discussant

## Abstract

The rise of Generative Artificial Intelligence (AI) tools such as Large Language Models (e.g., ChatGPT) has highlighted many potential application areas in gerontology, ranging from tools to support clinical decision support, facilitate communication, provide supportive services to older adults and family members and even address issues of social isolation and loneliness. Often technological solutions are developed without early and ongoing feedback by end users and other stakeholders. This symposium includes the presentation of distinct studies that examine the use of Generative AI for various gerontological areas based on experiences of the NIA funded Artificial Intelligence and Technology Collaboratories for Aging and other initiatives, and highlights ways to engage stakeholders in all design and implementation phases in order to facilitate co-creation of meaningful generative AI solutions. We provide a comprehensive discussion of both opportunities and challenges associated with the growth of Generative AI in gerontology and discuss clinical, ethical and practical implications. Additionally, we present ways to integrate Generative AI into traditional research and clinical practice in way that supplements rather than replaces traditional care, and minimizes the risk of bias.

